# Modification of the Tet-On regulatory system prevents the conditional-live HIV-1 variant from losing doxycycline-control

**DOI:** 10.1186/1742-4690-3-82

**Published:** 2006-11-09

**Authors:** Xue Zhou, Monique Vink, Ben Berkhout, Atze T Das

**Affiliations:** 1 Laboratory of Experimental Virology, Department of Medical Microbiology, Center for Infection and Immunity Amsterdam (CINIMA), Academic Medical Center of the University of Amsterdam, Meibergdreef 15, 1105 AZ Amsterdam, The Netherlands

## Abstract

**Background:**

We have previously constructed a doxycycline (dox)-dependent HIV-1 variant by incorporating the Tet-On gene regulatory system into the viral genome. Replication of this HIV-rtTA virus is driven by the dox-inducible transactivator protein rtTA, and can be switched on and off at will. We proposed this conditional-live virus as a novel vaccine approach against HIV-1. Upon vaccination, replication of HIV-rtTA can be temporarily activated by transient dox administration and controlled to the extent needed for optimal induction of the immune system. However, subsequent dox-withdrawal may impose a selection for virus variants with reduced dox-dependence.

**Results:**

We simulated this on/off switching of virus replication in multiple, independent cultures and could indeed select for HIV-rtTA variants that replicated without dox. Nearly all evolved variants had acquired a typical amino acid substitution at position 56 in the rtTA protein. We developed a novel rtTA variant that blocks this undesired evolutionary route and thus prevents HIV-rtTA from losing dox-control.

**Conclusion:**

The loss of dox-control observed upon evolution of the dox-dependent HIV-1 variant was effectively blocked by modification of the Tet-On regulatory system.

## Background

Live-attenuated SIV vaccines have proven the most effective approach to achieve protection against pathogenic challenge strains in the rhesus macaque model of AIDS [1-4]. However, persistent infection and low-level replication of the attenuated virus resulted in the selection of faster replicating variants that caused AIDS in some of the vaccinated macaques, particularly in neonates [5-9]. This vaccine approach is therefore considered unsafe for use in humans. We and others previously presented a conditional-live HIV-1 variant as a novel vaccine approach [10-14]. This HIV-rtTA virus does not replicate constitutively, but exclusively in the presence of the non-toxic effector doxycycline (dox). In HIV-rtTA, the viral transcriptional activator Tat and its TAR binding site were inactivated by mutation and functionally replaced by components of the Tet-On system for inducible gene expression [15-17]. The rtTA gene encoding the transcriptional activator was inserted in place of the *nef *gene, and the tet operator (*tetO*) DNA binding sites were introduced in the viral LTR promoter. The activity of rtTA is critically dependent on dox. This effector molecule binds to rtTA and triggers a conformational change that allows the protein to bind *tetO *DNA, resulting in activation of transcription and subsequent virus replication. The HIV-rtTA virus demonstrated dox-dependent replication not only *in vitro *in T cell lines and PBMCs [12], but also *ex vivo *in human lymphoid tissue [18]. Upon vaccination with this virus, replication can be temporarily activated by transient dox administration and controlled to the extent needed to elicit protective immune responses.

HIV-rtTA, like wild-type HIV-1, is subject to spontaneous evolution during replication due to error-prone reverse transcription and continuous selection pressure. We previously studied the evolutionary possibilities of HIV-rtTA in long-term cultures with dox, and demonstrated that the introduced components of the Tet-On system, which are essential for virus replication, were stably maintained in the viral genome. In fact, we observed mutations in both the rtTA gene and the *tetO *elements that significantly improved the replication capacity of the virus [19-22]. However, we also demonstrated that long-term replication of HIV-rtTA can result in virus variants that no longer depend on dox for replication [23]. This reduced dox-dependence was associated with a single amino acid substitution in the rtTA protein, either at position 19 (glycine to glutamic acid; G19E) or position 37 (glutamic acid to lysine; E37K). We subsequently developed an HIV-rtTA variant with alternative amino acids (G19F and E37L) at these positions that block the undesired evolutionary routes [23]. This novel variant showed improved genetic stability and did not escape from dox-control in long-term cultures with dox.

As a vaccine, replication of HIV-rtTA would be temporally switched on to induce anti-viral immune responses. Subsequent dox-withdrawal may impose alternative evolutionary pressure on the virus than long-term culturing with dox. Specifically, rtTA could evolve toward a reverse phenotype similar to the tTA transactivator of the Tet-Off system, which is constitutively active but inhibited by dox [15]. Such variants may have been actively counterselected in the previous evolution experiments with dox, but could appear in dox-washout experiments. We therefore followed HIV-rtTA evolution in multiple, independent cultures that were transiently activated by dox. The virus did indeed lose dox-control in a significant number of cultures following dox-withdrawal. We identified a typical amino acid substitution at position 56 in the rtTA protein that is responsible for the reduced dox-dependence. This rtTA variant indeed shows a reversed, tTA-like phenotype and was therefore never selected upon long-term culturing with dox. We developed a novel rtTA variant that blocks this undesired evolutionary route and thus improves the genetic stability and safety of the HIV-rtTA vaccine candidate.

## Results

### Evolution of HIV-rtTA after transient dox administration

**Figure 1:**
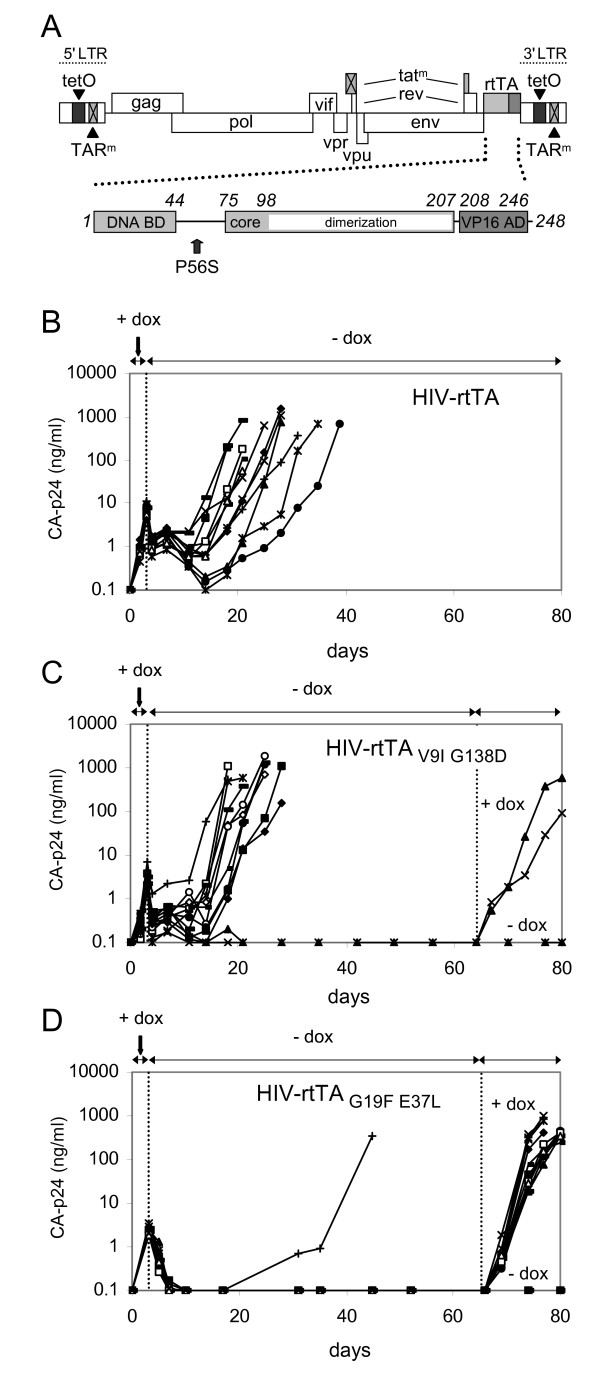
**Evolution of HIV-rtTA after transient dox administration**. (A) Schematic of the HIV-rtTA genome. The inactivated Tat-TAR elements (crossed boxes) and the introduced rtTA-tetO elements are indicated. rtTA is a fusion protein of the *E. coli *Tet repressor (TetR) and the VP16 activation domain (AD) of herpes simplex virus. TetR contains a DNA-binding domain (DNA BD) (amino acids 1–44) and a regulatory core domain (amino acids 75–207) with a dimerization surface. (B-D) Loss of dox-control in cultures of HIV-rtTA after transient activation. SupT1 cells were transfected with HIV-rtTA and cultured at 100 ng/ml dox (B), HIV-rtTA_V9I G138D _at 10 ng/ml dox (C), and HIV-rtTA_G19F E37L _at 1000 ng/ml dox (D). Each experiment was started with 12 independent cultures (different symbols represent different cultures). At day 3, dox was washed out and the cultures were continued with dox-free medium. The cultures in which the virus did not lose dox-control were split in two parts at day 64 (C) or day 66 (D) and dox was added to one of the samples. Virus production was monitored by CA-p24 ELISA on culture supernatant samples.

To test the genetic stability of HIV-rtTA (Fig. 1A) upon removal of the effector dox, we started 12 independent virus cultures in SupT1 T cells with dox (Fig. 1B). Viral replication resulted in the production of CA-p24 and the appearance of syncytia in all cultures. At day 3, we washed the cultures to remove dox, which resulted in silencing of viral replication as was obvious from the decrease in CA-p24 levels and the disappearance of syncytia in all cultures. However, CA-p24 levels started to increase again at day 10–20, and continued culturing resulted in high CA-p24 levels and formation of large syncytia. At the peak of infection, the virus was passaged onto fresh SupT1 cells and cultured without dox. All viruses were able to initiate a spreading infection, indicating that they had lost dox-control. Total cellular DNA with integrated proviruses was isolated from the cultures and the rtTA gene was PCR-amplified and sequenced. In all cultures, the virus had acquired the same point mutation (CCA to UCA) in the rtTA gene that resulted in a Proline to Serine substitution at position 56 (P56S).

Similar results were obtained with HIV-rtTA_V9I G138D_, an improved virus variant with two mutations in rtTA (V9I and G138D) that enhance transcriptional activity and dox-sensitivity [22]. The evolved viruses started to replicate without dox in 10 of the 12 cultures (Fig. 1C). Nine virus cultures acquired the P56S mutation, whereas one culture obtained the previously described G19E mutation that causes dox-independence [23]. In the two remaining cultures, CA-p24 levels stably decreased after dox removal and no viral replication was observed upon prolonged culturing. At day 64, these cultures were split and continued with and without dox. While the cultures without dox remained negative for CA-p24, spreading infections were apparent in the cultures with dox (Fig. 1C). Thus, the virus in these two cultures remained dox-dependent and can be readily reactivated.

### P56S mutation causes a tTA-like phenotype

**Figure 2:**
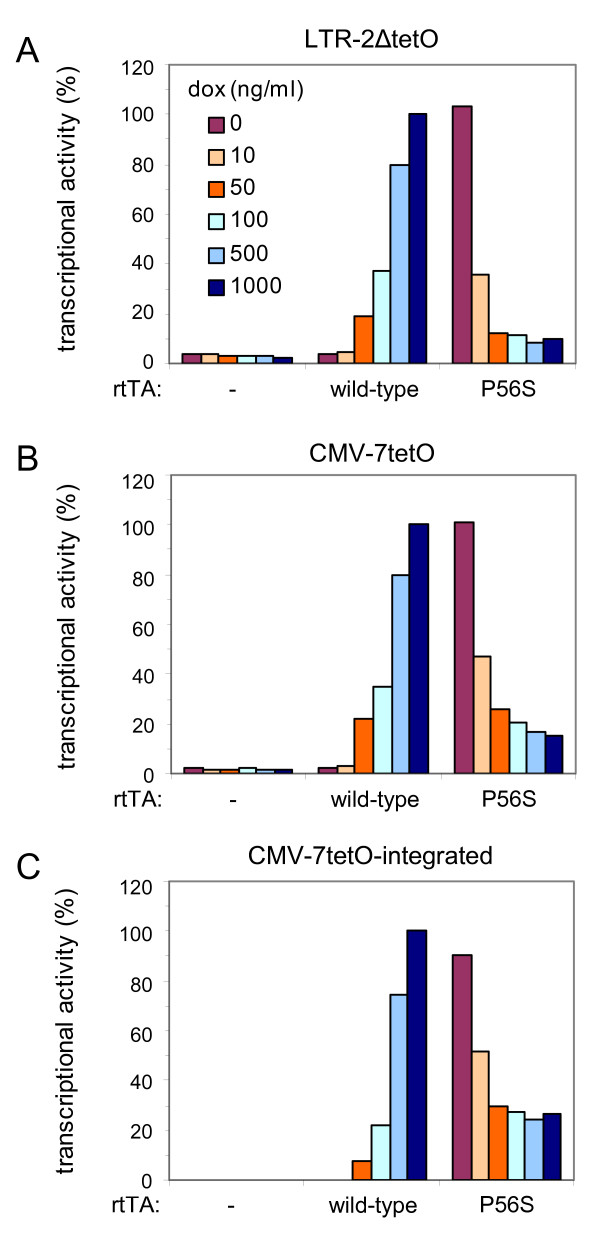
**The P56S mutation causes a tTA-like phenotype**. The activity of wild-type and P56S-mutated rtTA was measured in C33A cells transfected with a reporter plasmid carrying the firefly luciferase gene under the control of the viral LTR-2ΔtetO promoter (LTR-2ΔtetO; A) or under the control of a minimal CMV promoter coupled to an array of seven *tetO *elements (CMV-7tetO; B). Furthermore, rtTA activity was measured in HeLa X1/6 cells [25] that contain chromosomally integrated copies of the CMV-7tetO luciferase construct (CMV-7tetO-integrated; C). Cells were transfected with the indicated rtTA expression plasmid (both rtTA variants contain the F86Y and A209T mutations [19]) or pBluescript as a negative control (-), and a plasmid constitutively expressing *Renilla *luciferase to correct for differences in transfection efficiency. Cells were cultured with different dox concentrations (0–1000 ng/ml). The ratio of the firefly and *Renilla *luciferase activities measured two days after transfection reflects the rtTA activity. All values are relative to the wild-type rtTA activity at 1000 ng/ml dox, which was arbitrarily set at 100%.

The repeated selection of the P56S mutation in multiple, independent cultures strongly suggests its linkage to the observed loss of dox-control. To demonstrate that this amino acid substitution is indeed responsible for an altered rtTA phenotype, we cloned the P56S-mutated rtTA gene into the expression plasmid pCMV-rtTA and assayed its activity in a regular Tet-On system. The rtTA expression plasmid was transfected into C33A cells together with a reporter plasmid in which luciferase expression is controlled by the viral LTR-2ΔtetO promoter [20,21]. Transfected cells were cultured for two days at different dox concentrations. We subsequently determined the intracellular luciferase level, which reflects rtTA activity (Fig. 2A). Wild-type rtTA shows no activity without dox or with a low dox level (10 ng/ml), and its activity gradually increases at higher dox concentrations. In contrast, the P56S variant exhibits a very high activity without dox, and its activity is inhibited, instead of activated, by increasing dox concentrations. This phenotype is similar to that of the transcriptional activator tTA, which differs from rtTA by four amino acids, including an Alanine instead of Proline at position 56 [17]. The high activity of the P56S variant in the absence of dox explains its appearance in the dox-washout experiments, whereas its low activity with dox explains why we never observed this mutation in long-term cultures of HIV-rtTA in the presence of dox.

We also analyzed rtTA activity in C33A cells transfected with a luciferase reporter under the control of a minimal CMV promoter coupled to an array of seven *tetO *elements [24], and in HeLa X1/6 cells that contain stably integrated copies of this CMV-7tetO luciferase construct [25]. In both assays, we observed similar results as with the viral LTR-2ΔtetO promoter construct (Fig. 2B and 2C), demonstrating that the tTA-like phenotype of rtTA_P56S _is not dependent on the type of promoter, nor on the episomal or chromosomal state of the reporter gene.

### HIV-rtTA_G19F E37L _can lose dox-control by a P56S mutation

We have previously constructed an HIV-rtTA variant with the mutations G19F and E37L that prevent the virus from losing dox-control during long-term culturing with dox [23]. We now tested the stability of HIV-rtTA_G19F E37L _in the dox-washout experiment. This virus did lose dox-control in only one of the 12 cultures, and the remaining cultures did not show any replication in the absence of dox (Fig. 1D). Sequence analysis revealed that the single escape variant also acquired the P56S mutation. This result demonstrates that, although HIV-rtTA_G19F E37L _showed a lower tendency to lose dox-control than the original HIV-rtTA virus (Fig. 1B), the escape route at position 56 has to be blocked to further improve the genetic stability of the virus.

### Alternative amino acid at position 56 that poses a higher genetic barrier for viral escape

The P56S mutation is caused by a single nucleotide substitution (CCA to UCA). Such transitions occur at a higher frequency than transversions or multiple nucleotide changes during HIV-1 reverse transcription [26,27]. This mutational bias can strongly influence the course of virus evolution [28,29]. Accordingly, the undesired evolutionary route at position 56 may be blocked by the introduction of an alternative amino acid codon that requires multiple nucleotide changes for HIV-rtTA to lose dox-control. In fact, we have successfully blocked the escape routes at positions 19 and 37 by such mutations, demonstrating the effectiveness of this strategy [23]. To block all three observed escape routes of HIV-rtTA at the same time, the position 56 mutation should ideally be combined with the position 19 and 37 mutations. To identify suitable amino acid substitutions, we made rtTA expression plasmids with all possible amino acids at position 56 in combination with the G19F and E37L mutations, and assayed their activity in HeLa X1/6 cells.

**Figure 3:**
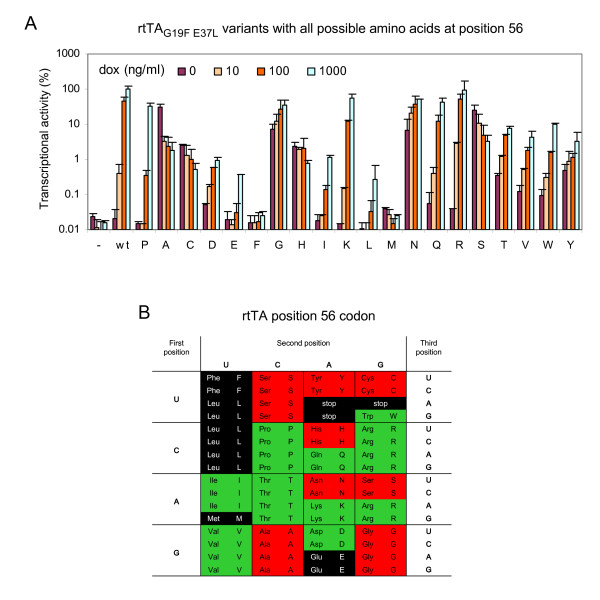
**Activity of rtTA**_G19F E37L _**variants with all possible amino acids at position 56**. (A) The activity of rtTA was measured in HeLa X1/6 cells, see Fig. 2 for details. All variants contain the G19F, E37L, F86Y and A209T mutations in combination with different amino acids at position 56. The wild-type rtTA (wt) with only the F86Y and A209T mutations was included as a control, of which the activity at 1000 ng/ml dox was arbitrarily set at 100%. Average values of two transfections are shown with the error bars indicating the standard deviations. (B) Codon table of rtTA_G19F E37L _variants with all possible amino acids at position 56. The corresponding codons of inactive variants are marked in black, dox-dependent variants are in green, and variants that are active without dox are in red. See the text for details.

The activity of these 20 rtTA variants varies considerably (Fig. 3A). Like the escape variant S, the A, C and H variants exhibit a tTA-like phenotype, since their activity is relatively high in the absence of dox and drops with increasing dox levels. Except for the F and M variants that are completely inactive, the other variants exhibit an rtTA phenotype, since their activity increases with a rising dox level. However, the basal and induced activities (at 0 and 1000 ng/ml dox, respectively) of these variants differ significantly. Because the L variant shows an rtTA phenotype with a very low basal activity, we introduced this variant into HIV-rtTA and tested viral replication in SupT1 T cells. This virus did not replicate without dox, but also not with dox (results not shown), indicating that the induced activity of the L variant (~ 0.3% of the wild-type rtTA activity at 1000 ng/ml dox) is not sufficient to drive HIV-rtTA replication. This result is in agreement with our observation (not shown) that the wild-type rtTA does not support viral replication at 10 ng/ml dox (~ 0.4% rtTA activity; wt in Fig. 3A) and rtTA_G19F E37L _does not support replication at 100 ng/ml dox (~ 0.4% rtTA activity; P variant in Fig. 3A). All these results suggest that the E, F, L and M variants with both their basal and induced activities lower than 0.4% will not support viral replication. We therefore present the codons corresponding to these amino acids and the stop codons in black (Fig. 3B). The basal activity of the A, C, G, H, N, S, and Y variants is higher than 0.4%. Since the corresponding HIV-rtTA viruses may replicate without dox, their codons are colored red. The remaining variants that show a low basal activity (< 0.4%) and a high induced activity (>0.4%) are expected to result in dox-dependent viruses, and their codons are marked green.

In the codon table, every change in row or column represents a single nucleotide substitution. Apparently, the only position 56 codon that preserves dox-dependence (green) and requires more than a single nucleotide mutation to be converted to a codon that allows replication without dox (red) is the AUA codon encoding an Isoleucine. However, the activity of the I variant at 1000 ng/ml dox is only 1% of the wild-type level (Fig. 3A), which may result in a poorly replicating virus that can hardly induce a protective immune response *in vivo*. The K and Q variants, which show a dox-dependent activity similar to the P variant (rtTA_G19F E37L_), require at least one nucleotide transversion to be converted to a dox-independent variant. It has been shown that transversions occur less frequently than transitions during HIV-1 reverse transcription [26,27]. Consistent with this, we did frequently observe the P56S mutation (CCA to UCA transition), but never the P56A mutation (CCA to GCA transversion), although both mutations cause a similarly high activity in the absence of dox (Fig. 3A). Therefore, introduction of an AAG (K) or CAG (Q) codon at rtTA position 56 may block the appearance of dox-independent virus variants upon dox-withdrawal.

### Blocking the loss of dox-control by a triple safety-lock rtTA variant

**Figure 4:**
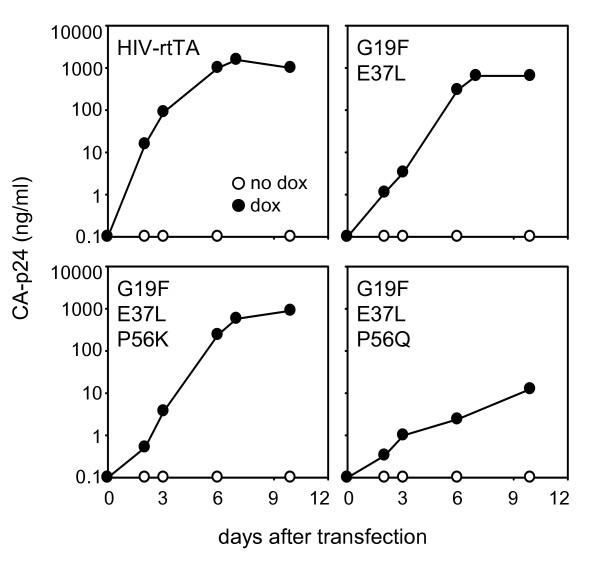
**Replication of HIV-rtTA**_G19F E37L _**variants with different amino acids at position 56**. SupT1 cells were transfected with 5 μg of HIV-rtTA molecular clones encoding different rtTA alleles, and cultured with or without 1 μg/ml dox. All rtTA variants contain the F86Y and A209T mutations. Virus replication was monitored by CA-p24 ELISA on culture supernatant samples.

**Figure 5:**
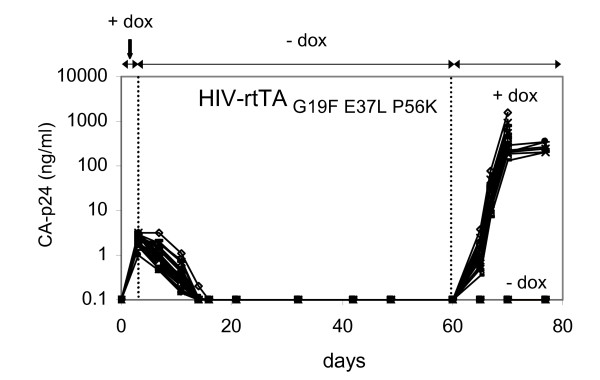
**Blocking the loss of dox-control by the triple safety-lock mutations**. SupT1 cells were transfected with HIV-rtTA containing the triple safety-lock mutations (HIV-rtTA_G19F E37L P56K_) at 1000 ng/ml dox and split into 24 independent cultures (different symbols represent different cultures). At day 3, dox was washed out and the cultures were continued with dox-free medium. At day 60, all cultures were split in two parts, and dox (1000 ng/ml) was added to one of the samples. Virus production was monitored by CA-p24 ELISA on culture supernatant samples.

We constructed HIV-rtTA molecular clones carrying the triple safety-lock mutations G19F, E37L and P56K or P56Q, and tested their replication in SupT1 T cells with and without dox (Fig. 4). Both viruses replicated in a dox-dependent manner. However, whereas replication of HIV-rtTA_G19F E37L P56K _was as efficient as the double safety-lock variant HIV-rtTA_G19F E37L_, HIV-rtTA_G19F E37L P56Q _replicated less efficiently. We therefore focused our studies on the HIV-rtTA_G19F E37L P56K _variant and tested the genetic stability of this virus in long-term cultures with dox and in dox-washout experiments. We started 24 long-term cultures with dox and tested virus replication in the presence and absence of dox at several time points. All virus cultures remained fully dox-dependent during 97 days of culturing, and sequence analysis revealed that the safety-lock mutations were stably maintained in all cultures (results not shown). To test the genetic stability of HIV-rtTA_G19F E37L P56K _after transient dox administration, we started 24 infections in the presence of dox (Fig. 5). Virus replication resulted in the production of detectable amounts of CA-p24 and the appearance of syncytia in all cultures. Upon dox-withdrawal at day 3, the CA-p24 level dropped and syncytia disappeared, and no sign of viral replication was apparent in any of the 24 cultures in the following months. At day 60, all cultures were split and continued with and without dox. While there was no viral replication in the cultures without dox, administration of dox did result in spreading infections, demonstrating that these viruses remained dox-dependent. This result also demonstrates that the undetectable CA-p24 level in dox-minus cultures is not due to loss of proviral genome or total silencing of the viral promoter. Therefore, replication of HIV-rtTA_G19F E37L P56K _remains dox-dependent in both long-term cultures with dox and transiently activated cultures, demonstrating that the triple safety-lock mutations at rtTA position 19, 37 and 56 effectively block the loss of dox-control.

## Discussion

Replication of the conditional-live HIV-rtTA virus is controlled by the incorporated Tet-On system. Previous studies demonstrated that the rtTA gene and the *tetO *elements are stably maintained in the viral genome to perform this essential function. Furthermore, these sequences are subject to random mutations introduced by the error-prone reverse transcriptase, and viral evolution has selected for optimized LTR-tetO promoter configurations and rtTA variants that greatly improved replication of HIV-rtTA [19-22]. Whereas most of these rtTA variants preserved strict dox-dependence of the virus, the acquisition of a G19E or E37K mutation resulted in virus variants that no longer depend on dox for replication [23]. All these evolution events were observed in long-term virus cultures in the presence of dox, which may not mimic the situation when HIV-rtTA is used as a vaccine. For a vaccine purpose, HIV-rtTA will only replicate temporally to induce an anti-viral immune response. Subsequent dox-withdrawal should block virus replication and prevent further evolution. However, this transient induction period may already have generated a viral quasispecies. Among such virus variants, there will be a strong selective advantage for those that are able to replicate without dox. To analyze the evolutionary possibilities of HIV-rtTA under such circumstances, we started multiple, independent virus cultures and transiently induced them by dox. A significant number of the cultures lost dox-control upon dox-withdrawal, and in nearly all cultures the virus acquired the P56S mutation. We demonstrated that this P56S mutation reverses the phenotype of rtTA, resulting in very high basal transcriptional activity that is gradually reduced by increasing dox concentrations. This tTA-like phenotype of rtTA_P56S _was observed in experiments with different cells and with different dox-responsive promoters that were either in an episomal or chromosomal state. Thus, a P56S-mutated rtTA variant can efficiently support viral replication in the absence of dox.

The transcriptional activator rtTA was originally identified as a tTA variant with the reverse phenotype concerning dox-control [16,17]. rtTA differs from tTA by four amino acid substitutions (E19G, A56P, D148E and H179R), of which the E19G and A56P combination is sufficient for the phenotypic reversal [17]. Our virus evolution studies demonstrate that the mutations at rtTA position 19 and 56 do indeed represent two possible evolutionary routes toward the loss of dox-control. The G19E mutation increases both the basal and the induced activities of rtTA, and was mainly observed in long-term virus cultures with dox [23]. The P56S mutation regenerates the tTA-like phenotype, i.e. the activity is inhibited, instead of activated, by dox. This phenotype explains why we did not observe P56S in long-term cultures with dox, but exclusively upon complete dox-removal. Amino acid variation at position 56 affects rtTA activity considerably (Fig. 3A). The phenotype of these variants can hardly be predicted by the chemical nature of the residue. This is probably due to the location of this residue in the helix connecting the DNA-binding domain and the effector-binding core domain (Fig. 1A). Effector binding in the core domain triggers a series of conformational changes, including a hinge-like movement of this helix and the attached DNA-binding domain [30,31]. The orientation of the DNA-binding domain determines the affinity of the protein for the *tetO *DNA sites and thus the transcriptional activity.

Our virus evolution experiments demonstrate that HIV-rtTA can escape from dox-control by an amino acid substitution in rtTA at position 19, 37 or 56. To generate a safe HIV-rtTA virus, all three evolutionary routes should be blocked. We have previously blocked the position 19 and 37 routes by mutations (G19F and E37L) that require multiple nucleotide changes to lose dox-control [23]. An additional safety-lock mutation (P56K) identified in this study further improves the genetic stability of HIV-rtTA. The novel virus variant with the triple safety-lock mutations replicated efficiently and in a dox-dependent manner. Importantly, it did not lose dox-control in either dox-washout experiments (Fig. 5) or in long-term cultures with dox. These studies demonstrate that the safety-lock mutations increase the genetic barrier for viral escape, thus making loss of dox-control less likely to occur. However, this block may not be absolute. Other strategies to improve the safety of HIV-rtTA as a conditional-live AIDS vaccine include deletion of non-essential parts of the viral genome to further attenuate the virus, e.g. the mini-HIV-1 approach [32,33]. Alternatively, a second regulatory mechanism such as the T20-dependent Envelope [34] may be incorporated to reduce the chance of viral escape [35].

## Conclusion

Evolution of the dox-dependent HIV-rtTA variant could result in the loss of dox control. This undesired evolutionary route was efficiently blocked by the development of a novel rtTA variant. We thus improved the genetic stability and safety of the HIV-rtTA vaccine candidate.

## Methods

### Virus cultures

The HIV-rtTA infectious molecular clone is a derivative of the HIV-1 LAI proviral plasmid [36] and was described previously [11,12]. HIV-rtTA used in this study contains the inactivating Y26A mutation in the Tat gene, five nucleotide substitutions in the TAR hairpin motif, the rtTA_F86Y A209T _gene [19] in place of the *nef *gene, and the LTR-2ΔtetO promoter configuration [20,21]. SupT1 T cells were cultured and transfected with HIV-rtTA molecular clones by electroporation as described previously [19]. The CA-p24 level in the cell-free culture supernatant was determined by antigen capture enzyme-linked immunosorbent assay (ELISA) [33].

The evolution experiment was started by transfection of 15 μg HIV-rtTA proviral plasmid into 1.5 × 10^7 ^SupT1 cells. Cells were split into 12 independent cultures and dox (Sigma D-9891) was added to initiate virus replication. Three days after transfection, dox was removed from the cultures by washing the cells twice with medium, each followed by a 30 min incubation at 37°C with 5% CO_2 _to allow release of dox from cells. Cells were subsequently resuspended in medium and cultured without dox. If virus replication was apparent as indicated by the formation of syncytia, the virus containing culture supernatant was passaged onto fresh SupT1 cells. Infected cell samples were used to analyze the proviral rtTA sequence.

### Proviral DNA analysis of evolved sequences

HIV-rtTA infected cells were pelleted by centrifugation and washed with phosphate-buffered saline. Total cellular DNA was solubilized by resuspending the cells in 10 mM Tris-HCl (pH 8.0)-1 mM EDTA-0.5% Tween 20, followed by incubation with 200 μg/ml of proteinase K at 56°C for 60 min and 95°C for 10 min. The proviral rtTA genes were PCR amplified with primers tTA1 (5'-ACAGCCATAGCAGTAGCTGAG-3') and tTA-rev2 (5'-GATCAAGGATATCTTGTCTTCGT-3'), and sequenced with the bigdye terminator cycle sequencing kit (Applied Biosystems).

### Construction of novel rtTA expression plasmids and HIV-rtTA variants

The pCMV-rtTA expression plasmid contains the improved rtTA_F86Y A209T _gene [19]. To introduce the P56S mutation, the proviral PCR product with this mutation was digested with XbaI and SmaI and used to replace the corresponding fragment in pCMV-rtTA. To generate rtTA variants with the G19F and E37L mutations and different amino acids at position 56, mutagenesis PCR was performed on pCMV-rtTA_G19F E37L _[23] with the sense primer random-rtTA-56 (5'-AAGCGGGCCCTGCTCGATGCCCTGNNKATCGAGATGCTGGACAGGC-3', with K corresponding to G or T, and N corresponding to G, A, T or C) plus the antisense primer CMV2 (5'-TCACT GCATTCTAGTTGTGGT-3'). Mutant rtTA sequences were cloned as ApaI-BamHI fragments into pCMV-rtTA_G19F E37L_. Novel rtTA sequences were cloned into the shuttle vector pBlue3'LTRext-deltaU3-rtTA_F86Y A209T_-2ΔtetO [19] using the XcmI and NdeI sites, and subsequently cloned into the HIV-rtTA molecular clone as BamHI-BglI fragments. All constructs were verified by sequence analysis.

### rtTA activity assay

pLTR-2ΔtetO-luc expresses firefly luciferase from the LTR-2ΔtetO promoter derived from the HIV-rtTA molecular clone [20,21]. pCMV-7tetO-luc, previously named pUHC13–3 [24], contains seven *tetO *elements located upstream of a minimal CMV promoter and the firefly luciferase gene. The plasmid pRL-CMV (Promega), in which the expression of *Renilla *luciferase is controlled by the CMV promoter, was used as an internal control to allow correction for differences in transfection efficiency. HeLa X1/6 cells are derived from the HeLa cervix carcinoma cell line and harbor chromosomally integrated copies of the CMV-7tetO firefly luciferase reporter construct [25]. HeLa X1/6 and C33A cervix carcinoma cells (ATCC HTB31) [37] were cultured and transfected by the calcium phosphate precipitation method as previously described [19]. C33A cells grown to 60% confluence in 2-cm^2 ^wells were transfected with 0.4 ng pCMV-rtTA, 20 ng pLTR-2ΔtetO-luc or pCMV-7tetO-luc, 0.5 ng pRL-CMV, and 980 ng pBluescript as carrier DNA. HeLa X1/6 cells were transfected with 8 ng pCMV-rtTA, 2.5 ng pRL-CMV, and 990 ng pBluescript. Transfected cells were cultured for 48 hours at different dox concentrations and subsequently lysed in Passive Lysis Buffer (Promega). Firefly and *Renilla *luciferase activities were determined with the Dual-Luciferase Reporter Assay (Promega) using a GloMax microplate luminometer (Promega). The expression of firefly and *Renilla *luciferase was within the linear range and no squelching effects were observed. The activity of the rtTA variants was calculated as the ratio of the firefly and *Renilla *luciferase activities, and corrected for between-session variation [38].

## Competing interests

The Academic Medical Center of the University of Amsterdam and the Technology Foundation STW applied for patents on the dox-dependent HIV-1 variant and on the novel rtTA variants.

## Authors' contributions

XZ and MV performed the experiments. XZ analyzed the data and drafted the manuscript. ATD and BB designed the experiments and revised the manuscript.
